# The zebrafish presomitic mesoderm elongates through compaction-extension

**DOI:** 10.1016/j.cdev.2021.203748

**Published:** 2021-09-28

**Authors:** Lewis Thomson, Leila Muresan, Benjamin Steventon

**Affiliations:** 1 Department of Genetics, University of Cambridge, Cambridge, UK, CB2 3EH; 2 Cambridge Advanced Imaging Centre, University of Cambridge, Cambridge, UK; 3 Present address: Department of Zoology, University of Cambridge, Cambridge, UK, CB2 3EJ

## Abstract

In vertebrate embryos the presomitic mesoderm becomes progressively segmented into somites at the anterior end while extending along the anterior-posterior axis. A commonly adopted model to explain how this tissue elongates is that of posterior growth, driven in part by the addition of new cells from uncommitted progenitor populations in the tailbud. However, in zebrafish, much of somitogenesis is associated with an absence of overall volume increase, and posterior progenitors do not contribute new cells until the final stages of somitogenesis. Here, we perform a comprehensive 3D morphometric analysis of the paraxial mesoderm and reveal that extension is linked to a volumetric decrease and an increase in cell density. We also find that individual cells decrease in volume over successive somite stages. Live cell tracking confirms that much of this tissue deformation occurs within the presomitic mesoderm progenitor zone and is associated with non-directional rearrangement. Taken together, we propose a compaction-extension mechanism of tissue elongation that highlights the need to better understand the role tissue intrinsic and extrinsic forces in regulating morphogenesis.

## 1 Introduction

A key process in early development is the progressive elongation of the embryo along the anterior-posterior axis. In vertebrates, this is coupled with the segmentation of the paraxial mesoderm into somites, that occurs in a clock-like manner from the anterior to the posterior as development continues (Cooke and Zeeman, 1976; Dequéant and Pourquié, 2008; Richardson et al., 1998). To ensure that the appropriate number of somites are generated upon completion of somitogenesis, this process must be balanced with the elongation of the presomitic mesoderm (PSM) (Gomez et al., 2008; Gomez and Pourquié, 2009). A dominant model for PSM elongation is that of posterior growth, based on the idea that cells are continually being added from progenitor populations in the tailbud and the widespread conservation of a common set of gene regulatory interactions in this process (Martin and Kimelman, 2009). However, fast-developing organisms such as zebrafish elongate their body axis in the absence of volumetric growth at the posterior end of the embryo (Bouldin et al., 2014; Steventon et al., 2016; Zhang et al., 2008). In addition, the tailbud population of neuromesodermal progenitors delay their contribution to the PSM until late stages of somitogenesis (Attardi et al., 2018). Therefore, it remains unclear as to how the zebrafish PSM elongates in the absence of posterior growth and progenitor addition.

Several recent studies have highlighted the importance of regulating the cell movements and fluidity of the PSM as cells leave the PSM progenitor domain in the tailbud (expressing *msgn1* (Fior et al., 2012)) and enter the mature PSM (expressing *tbx6* (Griffin et al., 1998)). We refer to these regions as the posterior PSM and the anterior PSM, respectively (given that they are two regions of one continuous tissue rather than separate tissues), and we use the term “PSM” to refer to the entire tissue - as used by Dequéant and Pourquié (2008); Dubrulle and Pourquié (2004); and Gomez and Pourquie (2009). Directional variability in the posterior PSM has been shown to be important for facilitating elongation by ensuring that the posterior flow of dorsal cells into this region is translated into a continuous, bilaterally symmetrical contribution to the anterior PSM (Lawton et al., 2013). This movement of cells from the posterior to anterior PSM is thought to be the result of active cell migration (Manning and Kimelman, 2015). Mongera et al. (2018) showed that there is a gradient of yield stress from posterior to anterior; with the posterior PSM being more “fluid-like” and showing more cell mixing than the anterior PSM. These authors proposed, using a 2D modelling approach, that a jamming transition between these two regions ensures that cell addition into the posterior PSM is translated into unidirectional elongation, rather than isotropic expansion. Importantly, these explanations were based on experiments using mid-somitogenesis embryos (10-14SS), in which cells are still being added to the posterior PSM from the dorsal medial zone through gastrulation movements. How the PSM continues to elongate at later somite stages has not yet been fully determined.

To answer the question of how the paraxial mesoderm elongates in the absence of posterior cell addition and growth, a long-term, multi-scale, three-dimensional approach is required. Through long-term 3D morphometrics and cell-tracking we show that elongation of the PSM is associated with overall tissue compaction. However, this “compaction-extension” is not driven by local cell intercalation, but through convergence of cells to the midline coupled with non-orientated cell intercalation.

## 2 Materials and methods

### 2.1 Animal husbandry and microinjection

Zebrafish embryos were raised in standard E3 media at 28°C. The following lines were used: Tüpfel Long Fin (TL), AB, AB/TL; and *h2b*::GFP. Embryos were staged according to Kimmel et al. (1995). mRNA (KikGR) was recovered from *E*. *coli* plasmid stocks using standard protocols and diluted to 100ng/μl in nuclease-free water, with phenol red added (0.05%) to help with visibility during microinjection. Embryos were microinjected (using pulled capillary needles) at the one-cell stage.

### 2.2 in situ hybridization chain reaction (HCR)

Embryos were manually dechorionated and fixed in 4% PFA in PBS (without calcium and magnesium) overnight at 4°C, then dehydrated in methanol and stored at -20°C. HCR was then performed using standard zebrafish protocol (Choi et al., 2018) and nuclei were stained with DAPI. The dehydration step was omitted when also staining membranes with phalloidin.

### 2.3 Imaging

For imaging fixed embryos, the tail was cut off and mounted in 80% glycerol. Live embryos were anaesthetised with tricaine and mounted whole in low melting-point agarose (1% in E3 media), covered in E3 media. The posterior part of the embryo was freed from agarose by cutting away the agarose with a microinjection needle, as described by Hirsinger and Steventon, (2017), which allowed normal development of the trunk and tail. All confocal imaging was performed on a Zeiss LSM 700 (inverted) and a Leica SP8 (inverted). Imaging was performed using 10x (air), 20x (air), or 40x (oil) objectives depending on the resolution required for analysis and on embryo size/mounting method. Live confocal imaging was performed with the use of a heated chamber (heated to 28°C). Live two-photon imaging was performed on a two-photon microscope (upright) with a 25x (water) objective and a heated chamber (heated to 28°C).

### 2.4 Image analysis

#### Imaris surfaces creation

**Figure 1:**
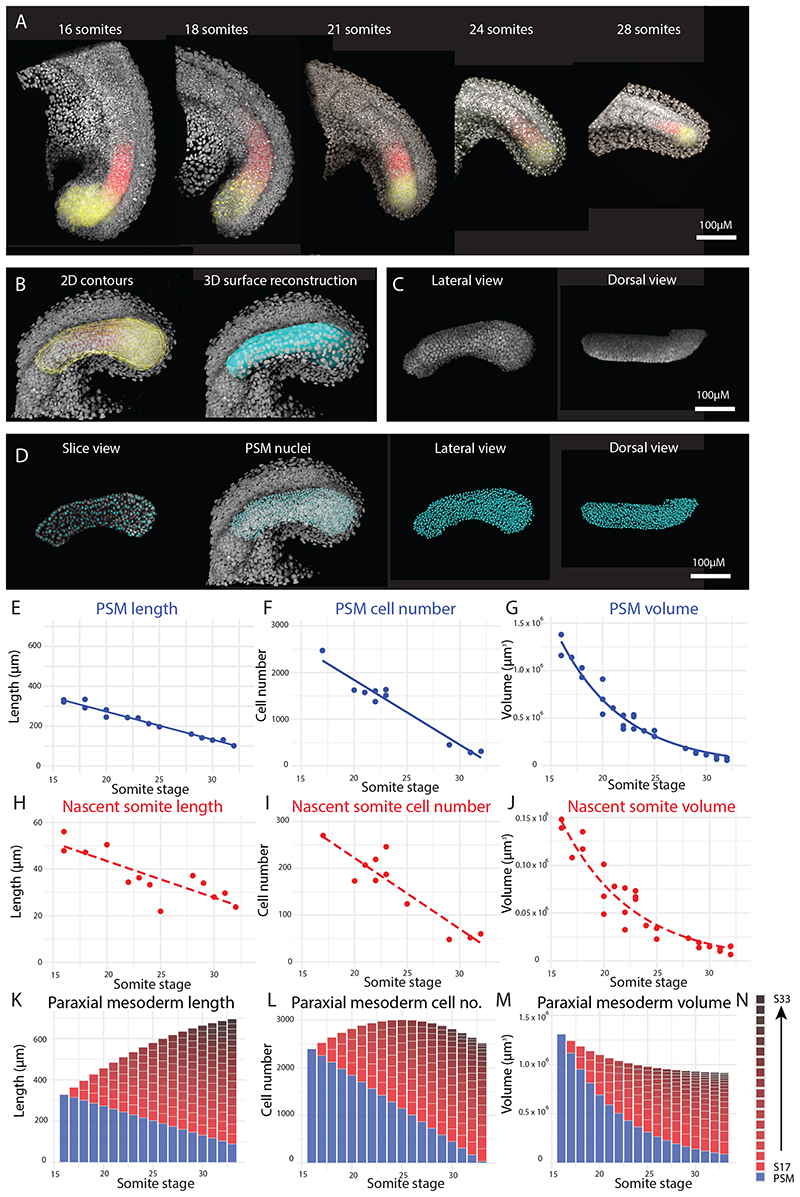
PSM elongation occurs in the absence of growth. (A) *in situ* hybridization chain reaction (HCR) was used to stain for *msgn1* (yellow) and *tbx6* (red), markers of the posterior and anterior PSM, respectively, from the 16 somite-stage to the end of somitogenesis. Nuclei were stained with DAPI (grey). (B) 2D contours (yellow outlines, left image) were manually drawn around the PSM at regular z-slices, up to the midline, to generate 3D surface reconstructions of the PSM (cyan, right image) and the nascent (i.e. most recently-formed) somite (Fig S1D) at each stage. (C) DAPI signal was isolated from each surface to show only nuclei in that tissue. (D) Spots were generated, marking the centre of each isolated nucleus (shown in slice view (left image) and 3D view (other images)), and providing cell number information. (E-J) The length, cell number, and volume of the PSM (E-G) and of each somite at its time of formation (H-J) was measured for a range of stages. Each point represents the PSM/nascent somite of a single embryo (n = 15 embryos for length data, n = 11 embryos for cell number data and n = 26 embryos for volume data). Possible trendline equations for each measurement were calculated in R, and AIC was used to determine the best statistical model (linear vs exponential). Solid trendlines (blue, PSM) indicate genuine change of one tissue over time, whereas dotted trendlines (red, nascent somites) indicate a trend based on separate tissues. The trendline equations are as follows (where *x* is somite stage): PSM length (E) = -14.1*x* + 155 (R^2^ = 0.97); PSM cell number (F) = -139*x* + 4,620 (R^2^ = 0.95); PSM volume (G) = 16,700,000e^-0.159*x*^ (R^2^ = 0.98); Nascent somite length (H) = -1.58*x* + 75 (R^2^ = 0.72); Nascent somite cell number (I) = -15.1*x* + 525 (R^2^ = 0.83); Nascent somite volume (J) = 1,630,000e^-0.151*x*^ (R^2^ = 0.94). (K-M) The above trendline equations were used to calculate how the length (K), cell number (L), and volume (M) of the paraxial mesoderm changed over time, by summing (for each stage) the current nascent somite and all previous nascent somite values to the current PSM value.

3D tissue reconstructions of paraxial mesoderm tissues (the PSM and nascent somite for each stage) were generated by creating “surfaces” in Imaris. This involved manually drawing “contours” around the tissue at regular z-intervals, which were then automatically translated into a 3D surface (Figure 1B; Figure S1A-C; Movie S1). Surfaces were created for one lateral half of the PSM, and the most recently-formed somite (i.e. the “nascent somite”) on the same side, at each stage. Imaris surfaces provided volumes of paraxial mesoderm tissues over time. A separate automatic surface was also generated of the posterior PSM/progenitor region, using *msgn1* HCR signal. This was useful for taking separate dimension measurements of the posterior and anterior PSM (see below).

#### Imaris spots creation

DAPI signal was isolated from the surfaces and used to generate a “spot” of each nucleus in paraxial mesoderm tissues. The estimated cell diameter used was 4 μm, and the minimum fluorescence intensity threshold was set to 0. These parameters gave the most accurate cell number estimates. This accuracy was validated by “masking” spots from the nuclei (to create a small black hole in each nucleus), then creating a new channel (given a different colour) with only DAPI signal inside the spots (to create a coloured dot in each nucleus). This method allowed us to check individual z-slices for the presence of one coloured dot in each nucleus. Estimated cell diameters below 4 μm led to multiple dots per nucleus (i.e. overestimation of cell number), while estimate cell diameters above 4 μm led to many nuclei with no dots (i.e. underestimation of cell number), as did setting any minimum fluorescence intensity threshold. Imaris spots provided cell numbers and 3D positions for paraxial mesoderm tissues over time.

#### Dimension measurements

**Figure 2:**
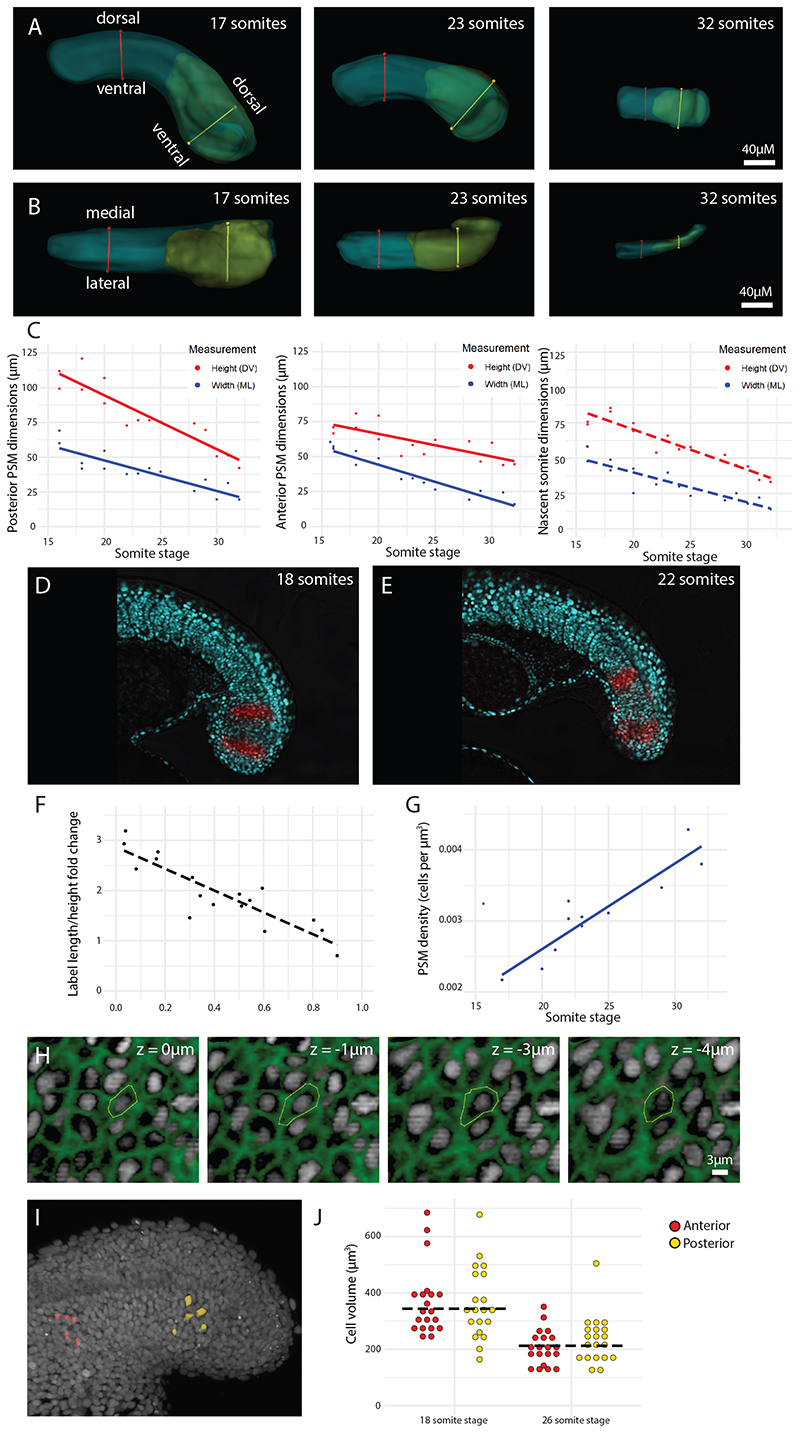
PSM convergence in height and width coincides with an increase in tissue density (A-B) Height (DV axis) (A) and width (ML axis) (B) measurements were taken of the posterior PSM (yellow line), anterior PSM (red line), and nascent somite (not shown) from the 16 somite-stage to the end of somitogenesis. (C) All height and width measurements show a decrease over the course of somitogenesis (n = 15 embryos). Solid trendlines indicate genuine change of one tissue (PSM) over time, whereas dotted trendlines indicate a trend based on separate tissues (nascent somites). The trendline equations are as follows (where *x* is somite stage): posterior PSM height = -3.88*x* + 172 (R^2^ = 0.84); posterior PSM width = -2.2*x* + 91.6 (R^2^ = 0.77); anterior PSM height = -1.62*x* + 98.7 (R^2^ = 0.58); anterior PSM width = -2.45*x* + 93.1 (R^2^ = 0.91); nascent somite height = -2.85*x* + 127 (R^2^ = 0.89); nascent somite width = -2.12*x* + 82.4 (R^2^ = 0.79). (D, E) Using a photoconvertible protein, dorsoventral stripes of cells in the PSM were photolabelled (red) and imaged over four somite-stages (n = 7 embryos). (F) The height and length of each label at the beginning and end of imaging was measured, along with the initial distance of the label from the posterior end of the PSM. These measurements were used to calculate a length/height ratio fold change over time, which is plotted over initial position AP position of the label (normalized from 0 to 1 between embryos, with 0 being the posterior end and 1 being the nascent somite posterior boundary). Dotted trendline equation: y = -2.17*x* + 2.87 (R^2^ = 0.78). (G) PSM density (cell number divided by tissue volume) increases over the course of somitogenesis. Trendline equation: y = 0.000121*x* + 0.000187 (R^2^ = 0.85). (H) Phalloidin-stained (green) tails of 18 somite-stage and 26 somite-stage embryos were used to measure cell volumes, by drawing contours around the cell membrane at every z-slice (z-interval: 1 μm). This was done for 5 randomly selected posterior cells and 5 randomly selected anterior cells per embryo (n = 10 embryos). (I) 3D reconstructions of posterior (yellow) and anterior (red) PSM cells, generated from manually-drawn contours. (J) Dotplot showing cell volumes between regions and between stages. Cell volumes were obtained from 3D cell reconstructions. Cell volumes are not significantly different between regions (t = -0.36, p = 0.72) but are significantly different between stages (t = 6.39, p < 0.001). The black dotted line represents the mean cell volume for each stage.

This was done manually in Imaris using “measurement points” and the 3D surfaces (described above) (Figure 2A-B). To measure length (anteroposterior (AP) axis) of the PSM, both the manual PSM surface and the automatic *msgn1* surface were used. The measurement was taken through the middle of the tissue, following the tissue curve by taking two measurements: one from the posterior medial face to the anterior face of the *msgn1* surface, and one from here to the anterior face of the PSM surface. This length measurement thus considered the tissue curve, as well as the fact that the surface is only one lateral half of the true tissue. For both width (mediolateral (ML) axis) and height (dorsoventral (DV) axis) measurements, separate measurements were taken for the posterior and the anterior PSM, and were taken at the halfway point (AP) of each region. While the ML axis keeps the same orientation between posterior and anterior PSM, the DV axis does not, in that the tail is curled ventrally. The two separate height measurements take this ventral curl into account. Somite measurements for each axis were also taken through the middle of the tissue.

#### Cell tracking

Automatic nuclear tracking in Imaris involves two steps: spot creation (as described above) and spot tracking, which requires a choice of tracking algorithm and a choice of parameters. After manually validating tracks from a range of algorithms and parameter sets (Figure S5), we used Autoregressive Motion (AM) as the tracking algorithm for all three movies, with the Gap Size parameter set to 3 for all movies. For movies M1 and M3, a Max Distance (MD) parameter of 5 was used, but for movie M2 an MD of 6 was used. Because M3 showed both long track durations (mean = 50 min) and high tracking accuracy, this movie was used as the main movie in tracking analyses, with the other two movies used as additional movies to check the generality of results.

## 3 Results

### 3.1 The zebrafish presomitic mesoderm decreases in volume as it extends

To better understand the tissue dynamics involved in zebrafish paraxial mesoderm elongation, a long-term, three-dimensional approach is required to determine the overall shape changes associated with elongation. We first made use of *in situ* hybridization chain reaction (HCR) to stain for the PSM markers *msgn1* and *tbx6* in embryos fixed at various stages of somitogenesis, together with DAPI to mark nuclei (Figure 1A). We focused on embryos past the 16 somite-stage to ensure that direct convergence of cells into the paraxial mesoderm from more lateral regions was no longer happening. Although the exact stage at which this process stops is not known, previous work has shown that cells can directly enter the somitic mesoderm of up to the 17^th^ somite, without having derived from the tailbud (Steventon et al., 2016). 3D reconstructions of the PSM were created by generating 2D contours around the tissue, at regular z-intervals, for each image (Figure 1B; Figure S1A-C; Movie S1). To obtain information about cell numbers over time, we created “spots” automatically using Imaris: the nuclei (DAPI signal) from each PSM/somite surface were isolated (Figure 1C) and a spot was generated for each nucleus (Figure 1D). Together, these measurements provided information about tissue lengths, volumes, and cell numbers over time.

Measurements were taken of the PSM and the most recently-formed somite (“nascent somite”) at each stage. This allowed us to measure changes to the PSM over time and the size of each somite at its time of formation. It also enabled us to calculate length, volume, and cell number values for the whole paraxial mesoderm (from the 16^th^ somite onwards) in a way that eliminated any growth or compaction happening in the somites after their formation. We did this by summing (for each stage) the current nascent somite and all previous nascent somite values to the current PSM value. In other words, we calculated the length, cell number, and tissue volume of the 16 somite-stage PSM from this stage until the end of somitogenesis, including the tissues (somites) it gave rise to, but excluding processes not relevant to PSM elongation.

Zebrafish PSM length (Figure 1E), cell number (Figure 1F) and volume (Figure 1G) all showed a reduction over time, as somites are continually being generated at a faster rate during these stages than any expansion or elongation of the PSM. Additionally, each nascent somite is smaller than the previous one, and so the somites display a similar gradual reduction in length (Figure 1H), cell number (Figure 1I) and volume (Figure 1J) as observed for the PSM. The above trend in length reduction (of both the PSM over time and of each nascent somite compared to the previous one) has been reported previously in zebrafish (Gomez et al., 2008; Schröter et al., 2008; Soroldoni et al., 2014), but, to our knowledge, cell numbers and tissue volumes have not previously been measured. These values then enabled us to calculate how the paraxial mesoderm elongates over-time (as described above). Rather than using specific measurements, trendline equations were calculated from the data − in order to obtain average values for each stage that had more than one data point. The results show that the length of the paraxial mesoderm increases considerably (~ 100%) throughout these stages of somitogenesis (Figure 1K). Taken together with the PSM measurements, this result shows that the PSM is elongating, but the length of each nascent somite specified is greater than the amount of elongation between somite stages, and so the length shows a gradual net decrease.

Paraxial mesoderm cell number does not change substantially over time, only showing a net increase of ~10% (Figure 1L). This net increase in cell number involves an initial, larger increase before a slight decrease. This apparent decrease in cell number could be due to cells leaving the paraxial mesoderm to contribute to other tissues. Lee et al. (2013) previously found that somite cells exit the paraxial mesoderm and contribute to fin mesenchyme, and our observations additionally show that PSM cells exit the paraxial mesoderm (suggesting that these cells contribute to fin mesenchyme) (Figure S2). Paraxial mesoderm volume decreases over time (even during the initial increase in cell number) (Figure 1M), indicating that the net increase in paraxial mesoderm cell number does not lead to volumetric growth.

Taken together, these results show that the paraxial mesoderm elongates considerably over time but that this is not the result of growth. While the number of cells does increase slightly, this does not cause tissue expansion − as the tissue *decreases* in volume over time. Therefore, paraxial mesoderm elongation is coupled with an overall volume decrease in zebrafish embryos.

### 3.2 Tissue convergence in the dorsal-ventral and medio-lateral axes is accompanied by a reduction in cell sizes

To test whether tissue deformation could be driving tissue elongation, measurements of the both the posterior and the anterior PSM were taken along both the dorsal-ventral (DV; Figure 2A; Movie S1) and medio-lateral axes (ML; Figure 2B; Movie S1). These 3D measurements showed that both height and width decrease over time by ~ 50% (Figure 2C). To determine whether anterior PSM thinning is actively occurring in that region of the tissue or the result of being progressively generated from a thinning posterior PSM, we used photolabelling and live imaging to follow how groups of cells deform over time. Embryos were injected at the one-cell-stage with mRNA for KikGR − a fluorescent protein that switches from green fluorescence to red fluorescence upon photoconversion with UV light (Habuchi et al., 2008). Dorsoventral stripes (across the full height of the PSM) were labelled in both the posterior PSM and anterior PSM (Figure 2D,E; Movie S2). The length (AP axis) and height (DV axis) of these labels were measured immediately after labelling, and again after 2 hours. By accumulating measurements from labels placed at different regions across the PSM, a clear trend is observed with increased convergence and extension observed in the most posterior region. The length-height ratio triples in posterior-most labels but stays constant in anterior-most labels (Figure 2F). Given that anterior PSM labels show very little deformation, this suggests that anterior PSM thinning is mostly the result of this tissue being progressively generated by a thinner posterior PSM at each preceding stage and supports the notion that the active region of deformation is within the posterior progenitor domain.

The result that the paraxial mesoderm shrinks in volume over time suggests that PSM density increases over time, pointing to a mechanism by which the posterior PSM compacts in DV and ML axes, to extend the whole tissue along the AP axis. This was found to be the case, by dividing the number of PSM cells by the PSM tissue volume to obtain a density measurement in terms of cells per μm^3^ (Figure 2G). This density increase is substantial (~ 80%), which suggests that cells must themselves decrease in volume to account for tissue-level compression. To test this, we fixed two batches of embryos (one at the 18 somite-stage, and one at the 26-somite stage, n = 5 embryos per batch) and stained the cell membranes with phalloidin to create 3D reconstructions of cells (Figure 2H) in both the posterior (Figure 2I; yellow) and anterior (Figure 2I; red) PSM. 3D reconstructions were generated as before, by drawing 2D contours at regular z-intervals (in this case, contours were drawn at every z-slice for which the cell was visible). The results show that within each stage, there was no difference between anterior PSM cell size and posterior PSM cell size (Figure 2J; t = -0.36, p = 0.72). However, there was a significant difference between stages (Figure 2J; t = 6.39, p < 0.001) − PSM cells of 26 somite-stage embryos (mean = 220 μm^3^) were smaller than those of 18 somite-stage embryos (mean = 367 μm^3^). Importantly, while cell sizes could be reducing due to cell divisions without subsequent cell growth, the number of cells in the paraxial mesoderm remains relatively constant over time (Figure 1L). Therefore, we can rule out the possibility that cell volume decrease over time is the result of cell division, and confirm that it is an active process of compaction. Together, these results demonstrate that the posterior PSM undergoes a decrease in volume, and an increase in cell density together with a reduction in cell volumes.

### 3.3 Generation of 3D cell tracking data

To determine the cell behaviours and movements underlying the tissue-level compaction-extension of the zebrafish PSM, individual cell 3D tracks are required. To obtain cell tracks, we live-imaged zebrafish embryos spanning the 14-26 somite stages (Figure S3). To draw meaningful conclusions from cell tracks, it is important to measure cell displacement with respect to appropriate frames of reference. The zebrafish tailbud is elongating and uncurling throughout most of somitogenesis, and so tracking PSM cells through absolute space will simply reflect the global tissue movement. Using Imaris imaging analysis software, a “reference frame” was placed at the end of the tailbud, at the DV and ML midline (Figure S4). The reference frame includes axes, which were orientated to match the three biological axes (x = AP, y = DV, z = ML) and adjusted every 5 frames for the full movie duration, for each movie. Imaris then automatically calculated the movement and rotation of the reference frame, using linear interpolation, for intermediate frames, and placed the reference frame appropriately for these frames. This allowed visualisation (of movies/tracks) to reflect the normalisation for global movement.

**Figure 3:**
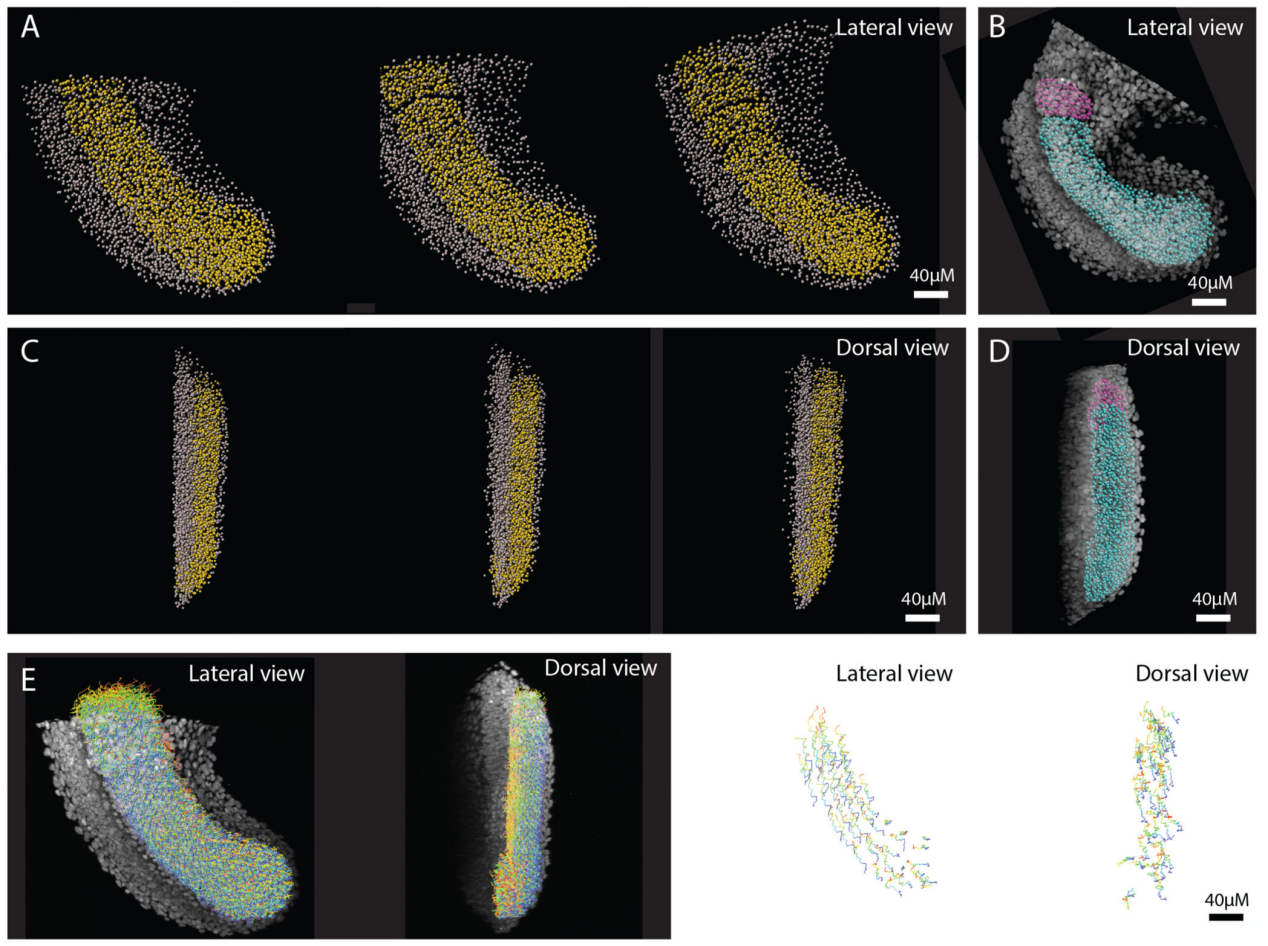
Paraxial mesoderm cell tracks. Tracks were generated of the whole tailbud and all non-paraxial mesoderm tracks were then manually removed. Paraxial mesoderm (PM) tracks (yellow spots) and non-paraxial mesoderm tracks (grey spots) shown at 0, 1, and 2 hours after imaging. (B) PM spots reconstructions (cyan: PSM & pink: nascent somite) from a similar-stage HCR image is shown for comparison/validation of selection accuracy. (C, D) The same images as above, but from a dorsal view: anterior is top, medial is left. (E) All PM tracks of full movie (colour-coded by time) superimposed over first frame image, shown for lateral and dorsal views (left images), and a sub-set of tracks shown in isolation, for lateral and dorsal views (right images).

Having chosen the best tracking algorithm and tracking parameters (see methods and Figure S5), we manually selected paraxial mesoderm tracks (Figure 3A,C yellow spots) from all cell tracks in the dataset (Figure 3A,C; grey spots). As in surface reconstructions (Figure 1), only one lateral half of the paraxial mesoderm was included − cells past the midline of the embryo were excluded. Because the images only contained nuclear signal, and no gene expression information (unlike the HCR images used for surface/spots reconstruction (Figure 3B, D; cyan and magenta spots)), it is likely that some notochord and neural progenitors are included in the tracks. However, these will constitute a negligible minority of cells, especially as any tracks that moved into the notochord or neural tube were excluded. The resulting cell tracks are shown colour-coded by time (Figure 3E; Movie S3).

### 3.4 PSM elongation is associated with non-directional cell rearrangement

**Figure 4:**
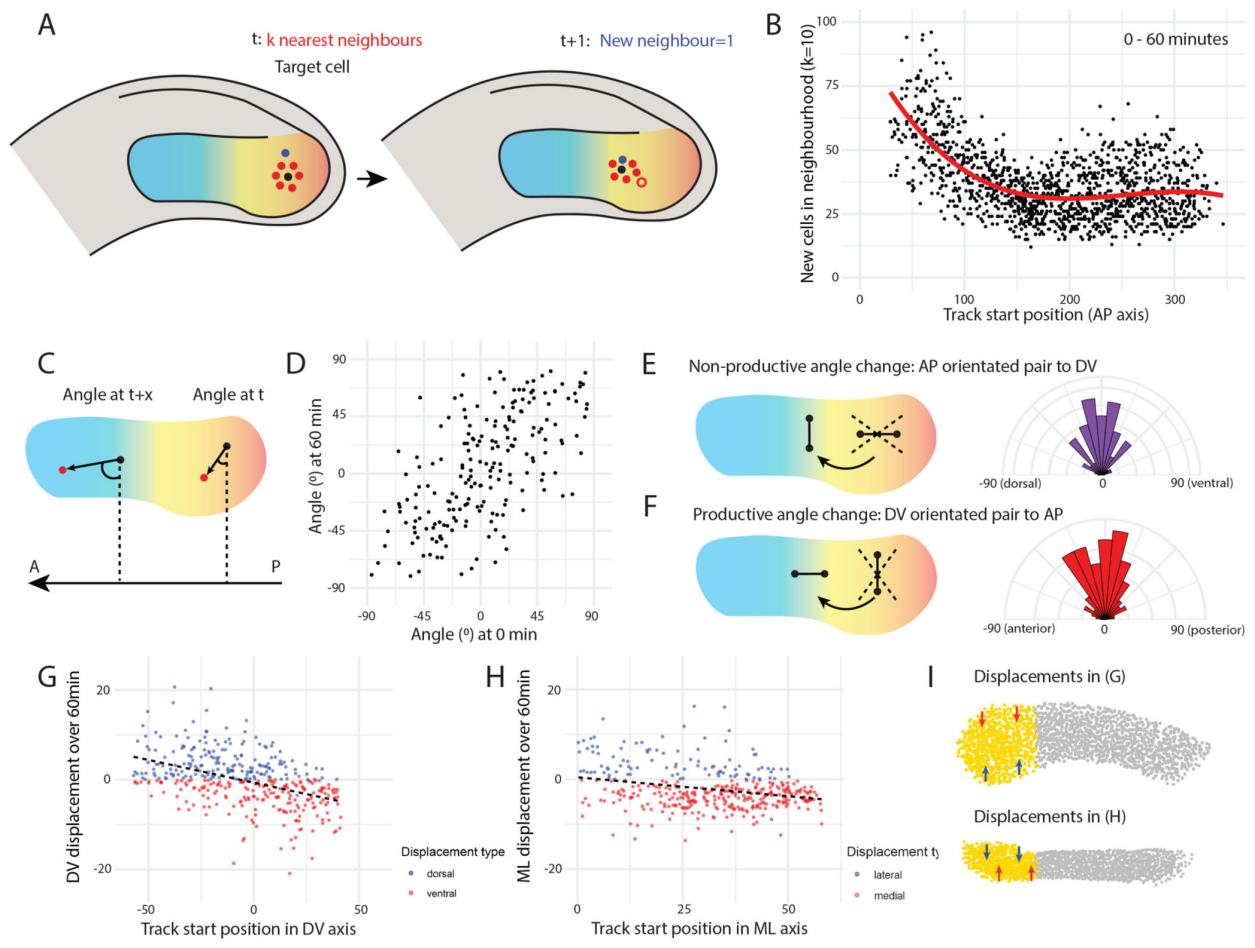
Posterior PSM cells drive convergent extension, but not via directional intercalation. (A) Analysis of neighbour exchanges. For each paraxial mesoderm cell (black), a neighbourhood was specified as a given number (k) of nearest neighbours (red cells). After a given time interval, the number of new cells (blue) that entered the target cell’s neighbourhood was calculated. In this example, one new cell joins the neighbourhood (k = 6) over time, with the cell that is no longer in the neighbourhood shown in red outline. (B) The number of new cells entering each target cell’s neighbourhood (k = 10) over 60 min is plotted against the initial AP position of the target cell, with 0 being the posterior end of the tail, and ~ 300 being the anterior-most cells. Each point represents a single cell (n = 1,348 cells from one embryo). The results show that more cell mixing occurs in the posterior PSM (C) Analysis of neighbour angle changes. For each posterior PSM cell (black), the angle between the vector to the nearest neighbour (red) and the AP axis was calculated at the initial timepoint (t). After a given time interval (t + x), the same vector angle was calculated between the two cells (regardless of whether they were still neighbours). 0°: cells lie perpendicular to the AP axis. 90°/-90°: cells lie parallel to the AP axis. An angle change from 0° to 90°/-90° would indicate strong directional intercalation of neighbouring cells. (D) Measured neighbour angle changes are shown for 60 min. Each point represents a cell pair (n = 201 cell pairs from one embryo), with the x-axis value providing the initial vector angle and the y-axis value providing the vector angle after 60 min. (E-F) Comparing the distribution of non-productive vs productive angle changes. (E) Those neighbour pairs that initially lay parallel to the AP axis (DV angle > -45° & < 45°) were selected, and the distribution of DV angle *changes* over 60 min is shown on the right. (F) Those neighbour pairs that initially lay perpendicular to the AP axis (AP angle > -45° & < 45°) were selected, and the distribution of AP angle *changes* over 60 min is shown on the right. The distributions of angle changes are not significantly different (Two-sample Kolmogorov-Smirnov test: D = 0.069652, p = 0.7141), suggesting that directional intercalation is not occurring. (G-I) Analysis of posterior PSM cell displacements (n = 1,161 cell tracks from one embryo). (G) Strong DV axis convergence over 120 min: dorsal cells (x > 0) move ventrally (y < 0, red) and ventral cells (x < 0) move dorsally (y > 0, blue). y = -0.1*x* − 0.67 (R^2^ = 0.2). (H) Weak ML axis convergence over 120 min: lateral cells (high x) move medially (y < 0, red) and medial cells (low x) move laterally (y > 0, blue). y = -0.08*x* + 0.45 (R^2^ = 0.08). (I) Schematic summarising displacements of posterior PSM cells (not to scale). Top view is lateral, showing DV convergence. Bottom view is dorsal, showing ML convergence.

We first set out to compare how cells differ in the rates of cell rearrangement as they transition from the posterior (Figure 4A; shaded red to yellow) to anterior PSM (Figure 4A; blue). To analyse this in 3D, we performed a nearest neighbour analysis that takes a given number *k* of nearest cells (neighbours) for each cell to form a neighbourhood for that cell at every frame. We then calculated the number of new cells that enter this neighbourhood over a given time *t* (Figure 4A).

The results of this analysis show that posterior cells exchange neighbours more than anterior cells (Figure 4B), which is consistent with measurements of mechanical properties of the tissue (Mongera et al., 2018). These results were consistent between small (*k* = 10) and large (*k* = 50) neighbourhoods, and between movies (Figure S6A). Given that an artificial increase in new neighbours might be caused in a region where cells are on average tracked for shorter duration (i.e. if average track duration was much lower in the posterior then new cell tracks might be interpreted as new neighbours), it was important to check that the observed trends were not simply an artefact of a possible correlation between track duration over track start position. However, there was no clear correlation between track duration and track start position (Figure S6B).

To qualitatively explore the directionality of cell movements within the PSM, we next measured the AP displacement of tracks over time. We used three reference frames along the paraxial mesoderm: the tailbud tip, the posterior end of the notochord proper, and the posterior boundary of the start-of-movie nascent somite (Figure S7). The results show that, relative to the tailbud tip or the posterior notochord, almost all cells displace anteriorly (Figure S8A,B). However, relative to the nascent somite, almost all cells displace posteriorly (Figure S8C). These results highlight the difficulty of defining movement as anterior vs posterior in an elongating tissue. While it is certainly the case that cells disperse along the AP axis, defining this dispersal as anterior vs posterior is purely subjective, based on which end of the embryo displacement is measured relative to. Given that the anterior end of the tissue/embryo is equally valid as a reference point as the posterior end is, there is no reason to define the AP dispersal as anterior movement.

During gastrulation, convergent extension of the paraxial mesoderm involves neighbouring cells undergoing directional intercalation; rearranging to line up along the axis of elongation (Keller et al., 2000). To determine whether directional intercalation is involved in zebrafish PSM elongation at later stages, we measured angle changes between neighbours, relative to the AP axis, over time. This involves taking the nearest neighbour for each cell at the start of the movie, calculating the vector between those two cells, and measuring the angle between this vector and the AP axis. Then, after a specified time-period *t*, the same two cells are taken (regardless of whether they are still neighbours), and their vector-angle relative to the AP axis is measured again (Figure 4C). In this way, the orientation of cell pairs along the AP axis can be measured over time, to test if neighbours are aligning with each other along the axis to drive elongation. In a situation where cells are directionally rearranging to drive convergence and extension along the AP axis, it would be expected, irrespective of the initial angle of nearest neighbours, that cell pairs should converge towards either 90°C or -90°C. Interestingly, while there is substantial rearrangement happening between neighbour pairs, this rearrangement does not appear to suggest directional intercalation. To examine this further, we separated cell pairs into those that initially lay parallel to the AP axis versus those that initially lay perpendicular to the AP axis. We then compared the distribution of angle *changes* over 60 minutes between these two groups. If directional intercalation was occurring, we would expect that those cell pairs that initially lie parallel to the AP axis should not change their neighbour angle, whereas those cell pairs that initially lie perpendicular to the AP axis should change their neighbour angle substantially. The results show that the distributions of angle changes (Figure 4E,F) are not significantly different (Two-sample Kolmogorov-Smirnov test: D = 0.069652, p = 0.7141). In other words, “non-productive” angle changes are just as common as “productive” angle changes. This strongly suggests that cell pairs are not undergoing directional intercalation to line up along the AP axis.

As the morphometric data showed convergence to the midline at the tissue level (Figure 2C), and photolabels confirmed this for the DV axis (Figure 2F), we next measured track displacements in the DV and ML axes in the posterior PSM. All tracks that began, in the first frame, between the tailbud tip and the notochord proper (i.e. posterior PSM tracks) were selected for analysis. For both DV and ML analyses, the tailbud tip reference frame was used, as this was the best reference frame to give a constant, correct midline for this region. The results show that posterior PSM cells do converge in the dorsoventral axis, with ventral cells displacing dorsally and dorsal cells displacing ventrally (Figure 4G). ML displacement was also observed, although to a lesser degree (Figure 4H) − as would be expected from previous tissue measurements showing a greater absolute decrease in height than in width. These displacements are summarised in Figure 4I (arrows not to scale).

## 4 Conclusions and Discussion

We propose that the zebrafish PSM elongates via a novel form of convergent extension, which we term “compaction-extension”. In the case of classical convergent extension during gastrulation, directional intercalation between neighbouring cells causes a thinning and lengthening of the tissue (Keller et al., 2000). We propose that, in the zebrafish PSM during mid-to-late somitogenesis stages, thinning is caused by compaction of the tissue, which partly results in tissue lengthening and partly results in cell shrinkage. This proposal is based on the following observations: firstly, elongation is associated with a decrease in tissue volume, with a concomitant increase in cell density and a decrease in cell volumes. Secondly, cells converge to the midline in both DV and ML axes, while dispersing along the AP axis. Finally, elongation occurs in the absence of directional cell rearrangement. An absence of directional intercalation between neighbours has been observed in zebrafish through following individual transitions in boundary contacts over short time-scales (Mongera et al., 2018). In addition by tracking cells and subtracting their movements from the average movements of neighbouring cells, it has been previously shown that the posterior PSM displays a disordered cell movement (Lawton et al. (2013)). Here, we confirm and extend these findings by showing that irrespective of their initial angle in 3D, cells do not show any evidence of orientating themselves along the AP axis. Nevertheless, it still may be the case that minor biases in the orientation of cell rearrangement, below the levels of statistical significance obtainable from our dataset, are sufficient to aid the convergence and extension of the PSM. However, taken together with previous findings, these results support a model in which non-directed cell movements are coupled with DV and ML convergence as the PSM compacts and extends along the AP axis.

That PSM elongation in zebrafish is associated with non-directional cell rearrangements reflects closely what has been observed in avian embryos (Bénazéraf et al., 2010). However, in the random motility model of avian PSM elongation, an important component is a density gradient along the AP axis. In this model, a low density of cells in the posterior (versus a high density of cells in the anterior) ensures that cell addition from the node causes the PSM to expand posteriorly. In zebrafish, there is no AP density gradient - instead, the posterior PSM appears to be more fluid-like due to decreased cell-cell adhesion (Mongera et al., 2018). This suggests that while an AP gradient in PSM tissue fluidity is conserved across vertebrates, the mechanism responsible for this is different among species (cell density in chick vs cell-cell adhesion in zebrafish). Alternatively, it could be that compaction-extension is a feature of tailbud stages of somitogenesis. Further studies in avian embryos measuring cell density over longer time periods − and including tailbud stages − would help answer these questions.

Although we show that the increase in PSM density is associated with a decrease in cell volumes, we do not know the causal relationship between these processes. Particularly, whether the mechanism is internal (i.e. the PSM is compacting from within due to cells contracting) or external (i.e. the PSM is compressed from outside, causing cells to shrink). A potential parallel for an internal mechanism of compaction-elongation could be mesenchymal cell condensation, as has been well studied in the context of skeletal (Hall and Miyake, 2000) and tooth (Svandova et al., 2020) development. If the PSM was being externally compressed (either by outer PSM cells, or by other tissues), this would likely lead to a mixture of high cell displacement, non-directional neighbour rearrangements, and cell shrinkage − all of which are happening in the zebrafish PSM. A likely mediator of the balance between tissue intrinsic cell movements and extrinsic forces is the extracellular matrix (ECM). Both PSM elongation and somitogenesis are dependent on integrins and fibronectin in zebrafish (Dray et al., 2013; Jülich et al., 2009), and appropriate re-modelling of the fibronectin matrix is mediated by the cell adhesion molecule Cadherin2 during somite formation (Jülich et al., 2015). Understanding how such mechanisms operate at the boundary of PSM as cells transit through the tissue will likely reveal new insights into the mechanisms of tissue elongation.

In addition to tissue-ECM interactions, it is likely that forces will be acting between adjacent tissues to help coordinate multi-tissue morphogenesis during body axis formation. Indeed, frictional forces acting between the notochord and PSM have been shown to be important for shaping the chevron-like shape of somites as they mature (Tlili et al., 2019), and anterior notochord expansion due to vacuolation is required for the continued elongation of the somites (McLaren and Steventon, 2021). How such multi-tissue interactions operate within the tailbud of amniote embryos has been recently addressed in a study that demonstrates a key role for PSM expansion in timing the entry of new cells to the posterior progenitor domain (Xiong et al., 2020). Taken together, these studies reveal posterior body axis elongation to be a tractable model system to better understand the role of multi-tissue mechanical interactions in morphogenesis. This work further enables this by providing a quantitative understanding of the tissue shape changes and cell rearrangements associated with PSM compaction-extension.

## Supplementary Material

Movie S1

Movie S2

Movie S3

Supplementary Figures

Supplementary
