# Supplementary material for: The zebrafish presomitic mesoderm elongates through compaction-extension
Source: Cells Dev. Author manuscript; Available in PMC 2022 May 11. (PMC7612712; doi:10.1016/j.cdev.2021.203748)
Supplement: Supplementary Figures [file EMS140703-supplement-Supplementary_Figures.pdf]

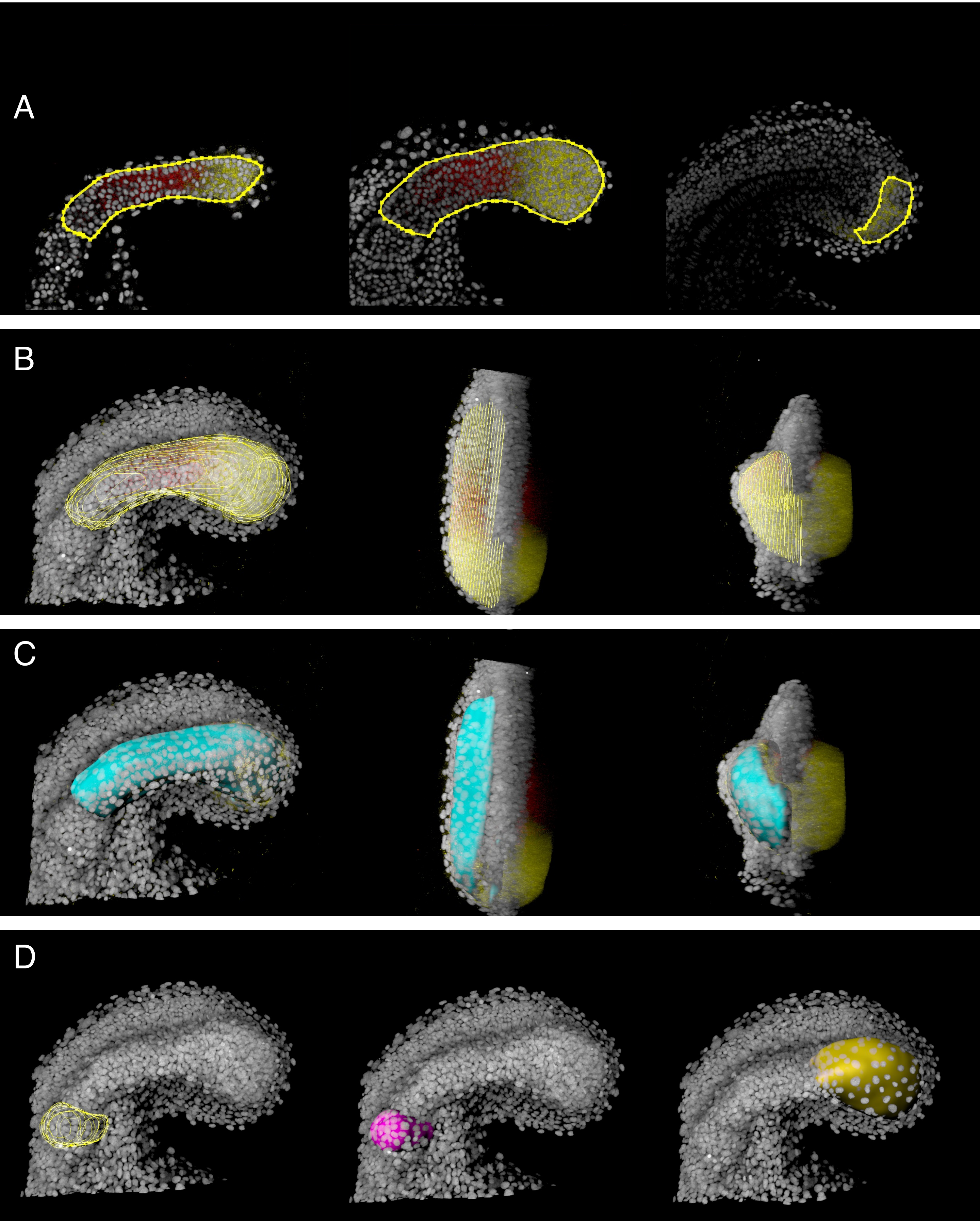

Figure S1

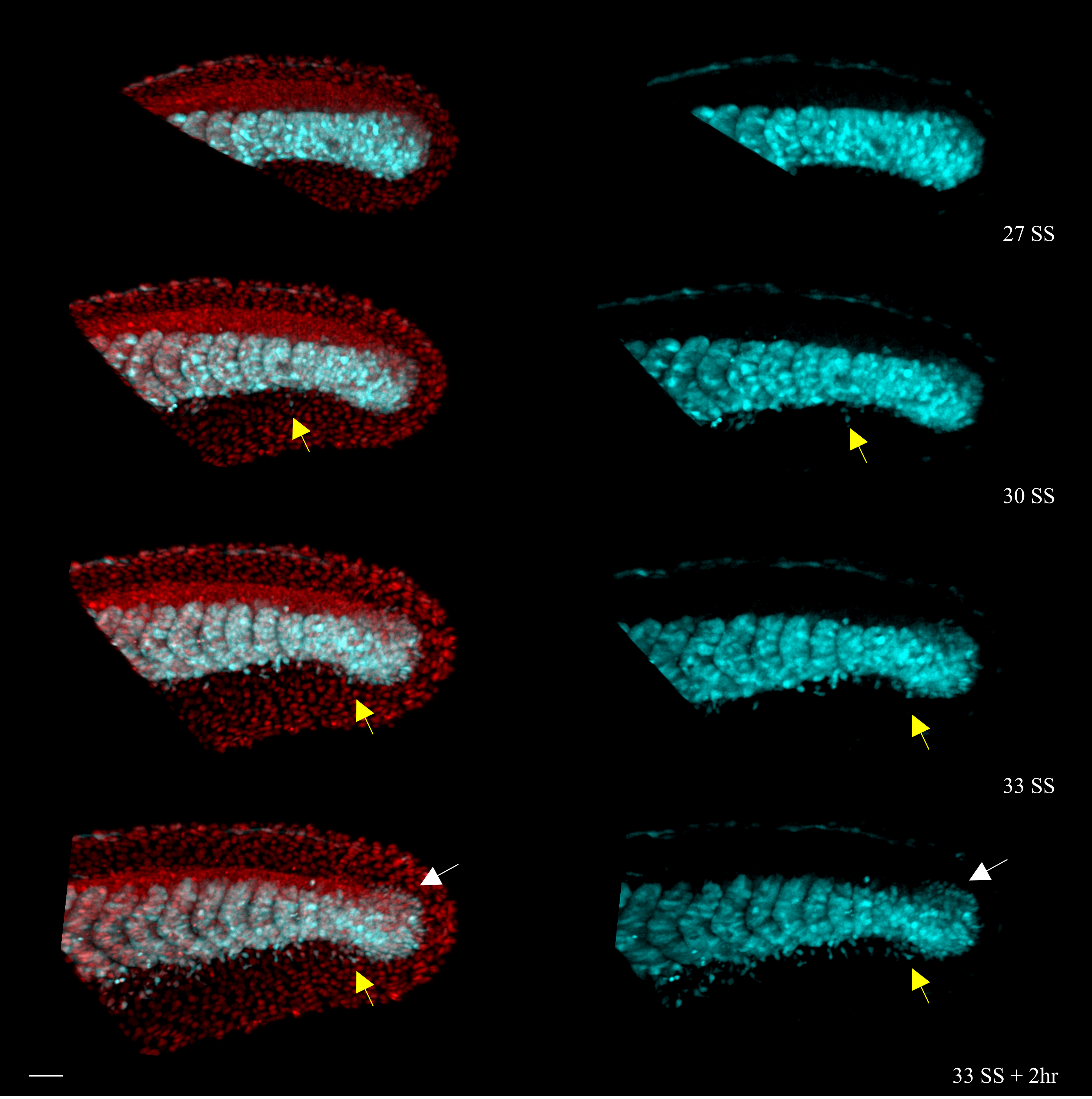

Figure S2

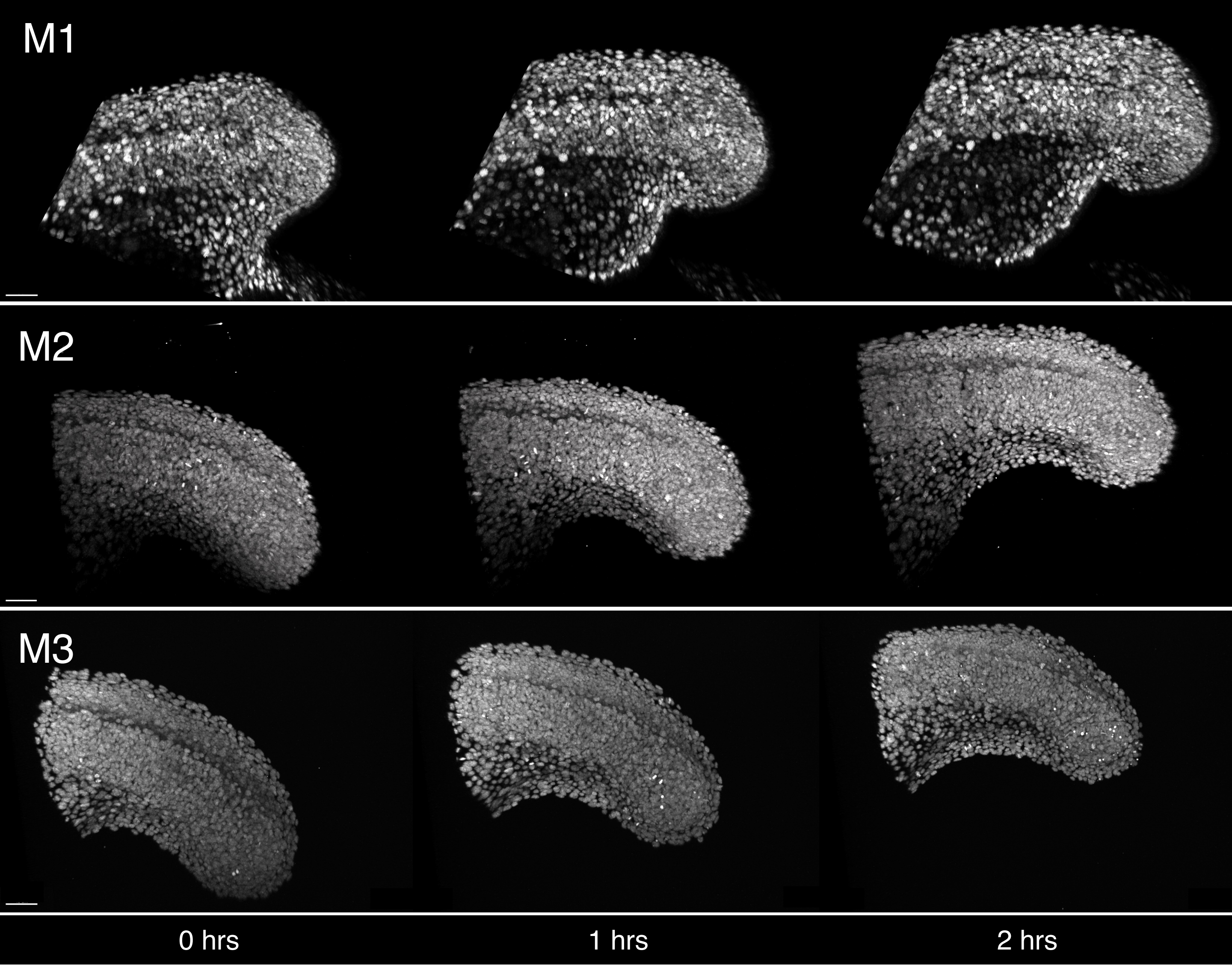

Movie	Somite-stages	Frame interval (s)	Nuclear label
M1	14 - 20	70	*h2a::mCherry*
M2	18 - 22	180	*h2b::GFP*
M3	22 - 26	150	*h2b::GFP*

Figure S3

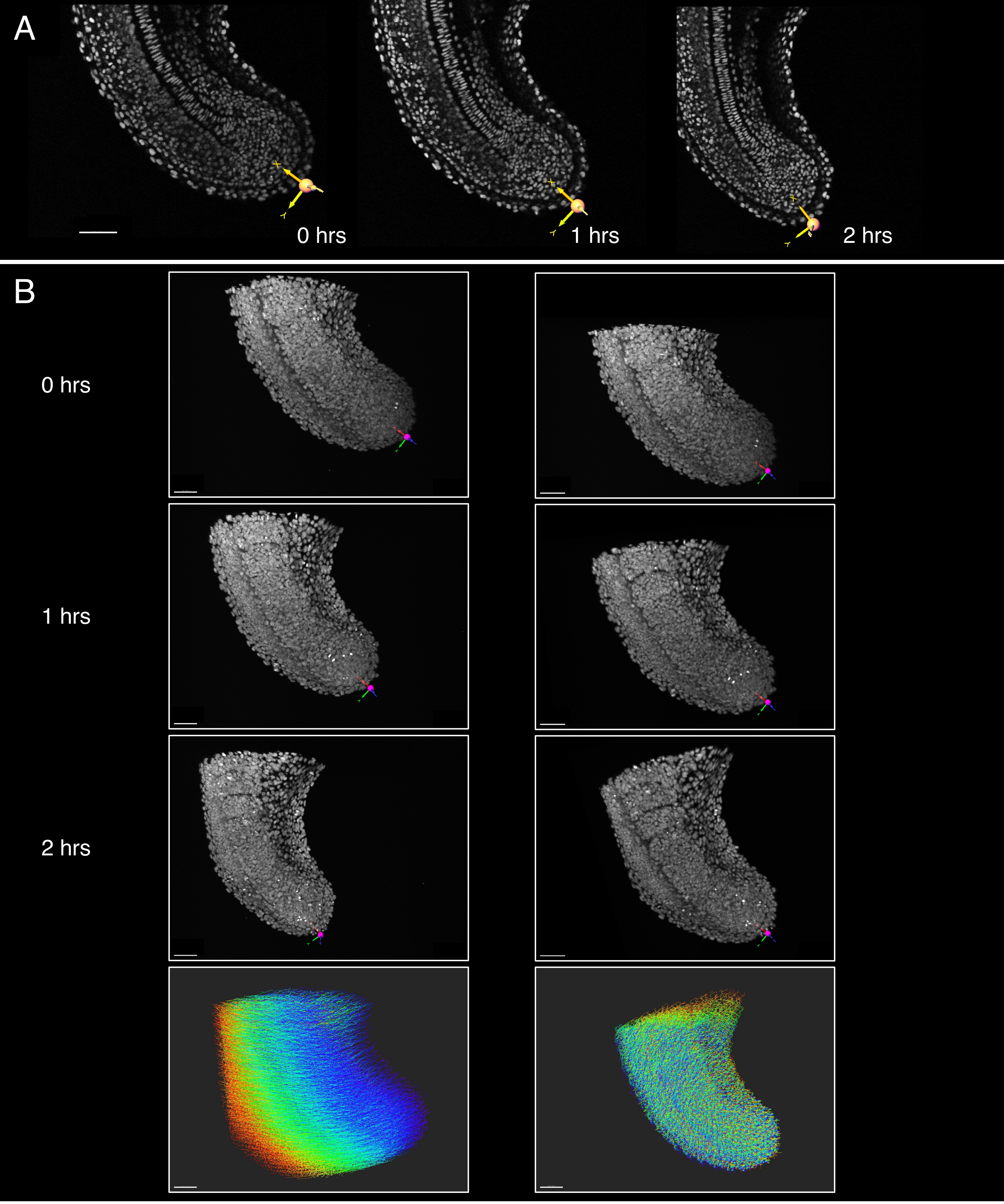

Figure S4

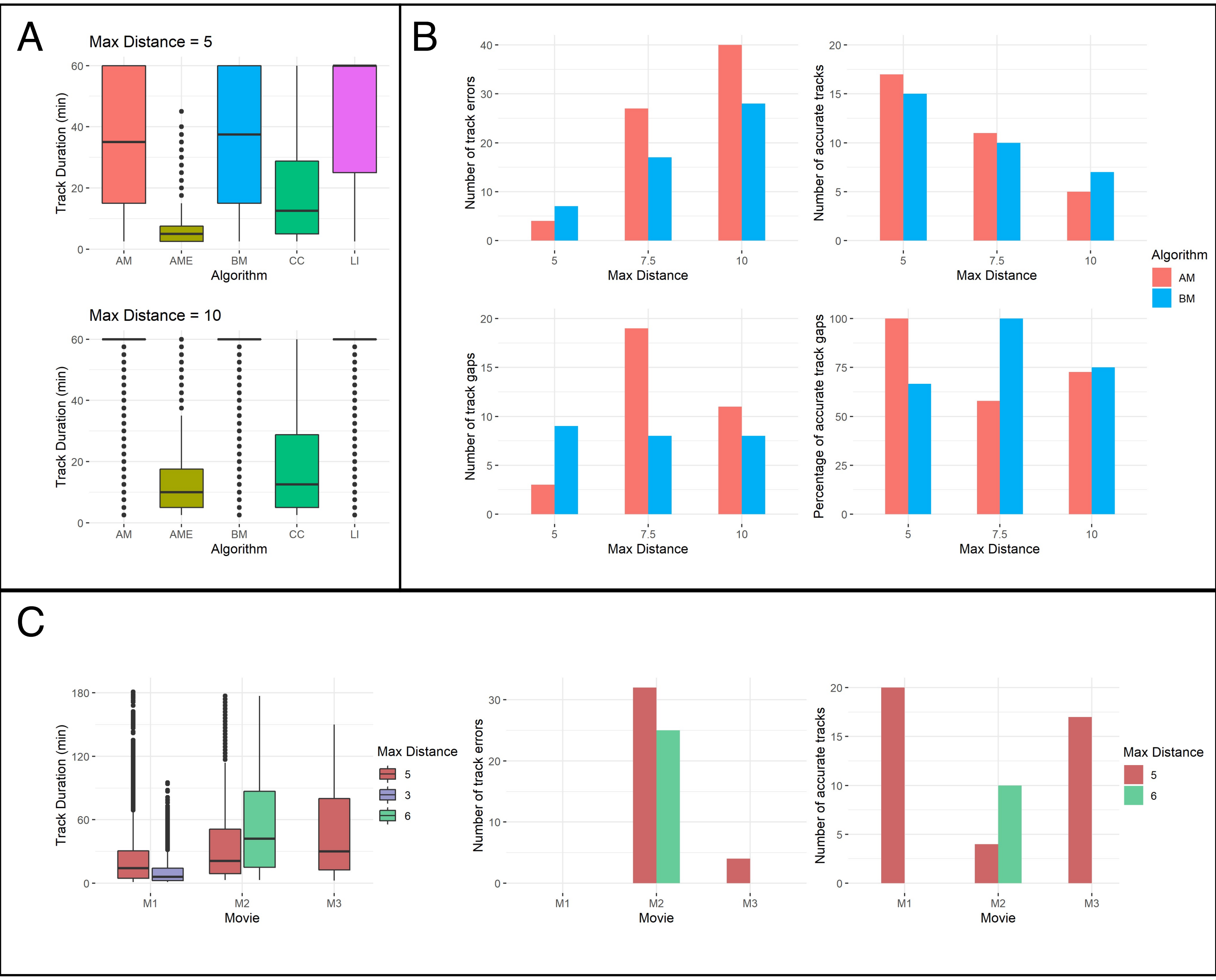
A
Max Distance = 5
Track Duration (min)
Algorithm
AM
AME
BM
CC
LI
Max Distance = 10
B
Number of track errors
Number of accurate tracks
Number of track gaps
Percentage of accurate track gaps
Max Distance
Algorithm
AM
BM
C
Track Duration (min)
Movie
M1
M2
M3
Max Distance
5
3
6
Number of track errors
Number of accurate tracks

Figure S5

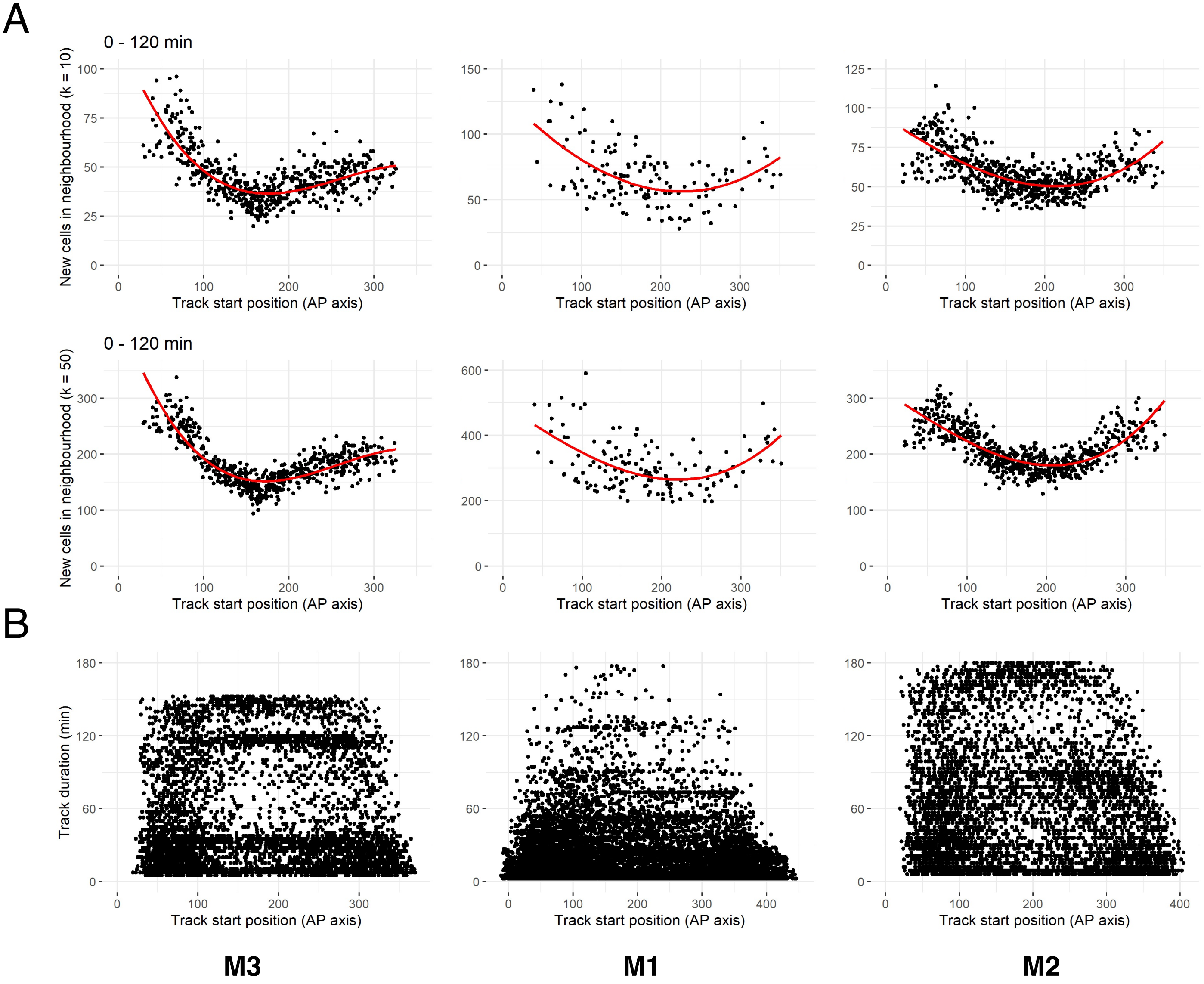
A
0 - 120 min
New cells in neighbourhood (k = 10)
Track start position (AP axis)
0 - 120 min
New cells in neighbourhood (k = 50)
Track start position (AP axis)
B
Track duration (min)
Track start position (AP axis)
M3
M1
M2

Figure S6

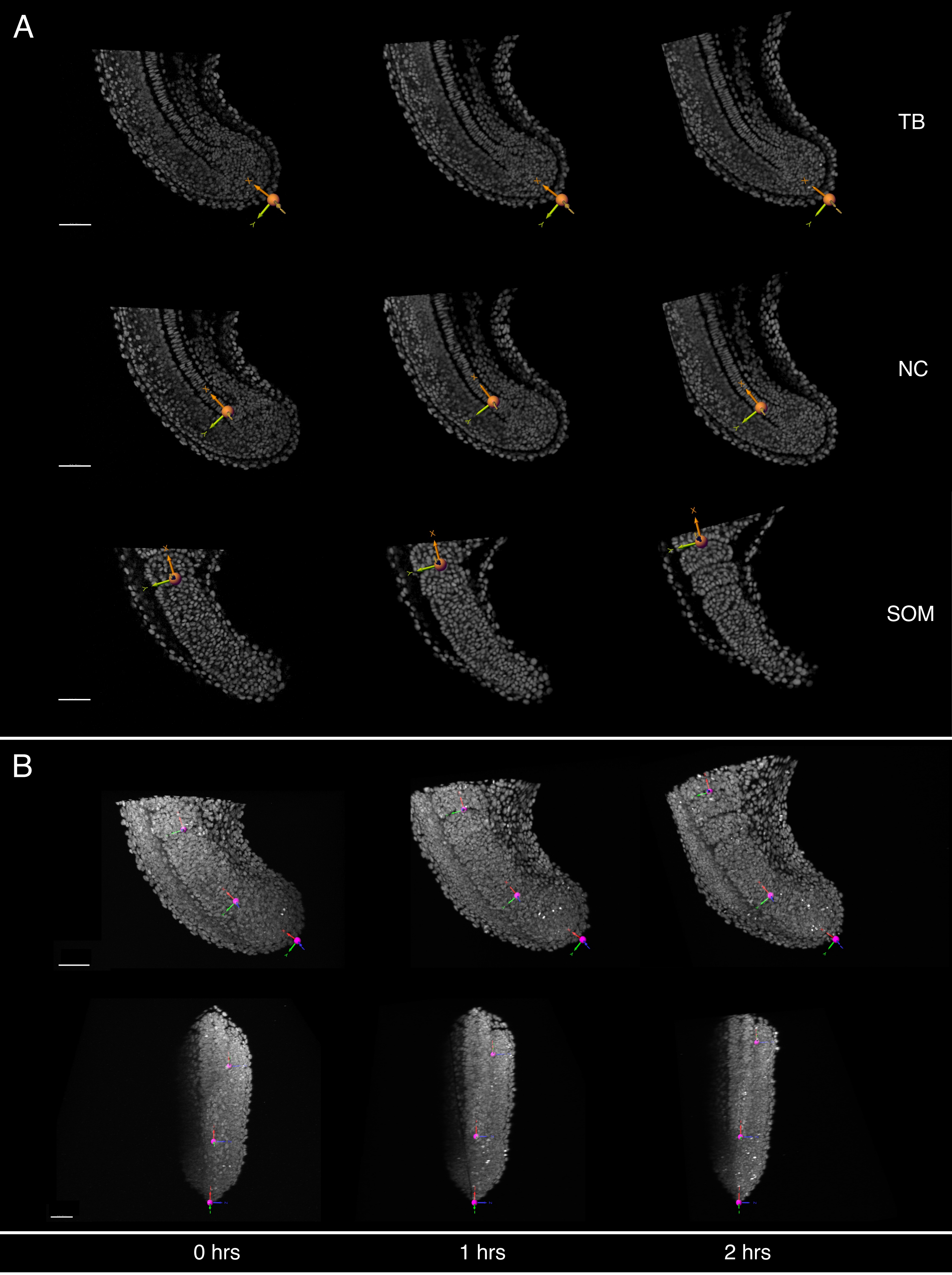

Figure S7

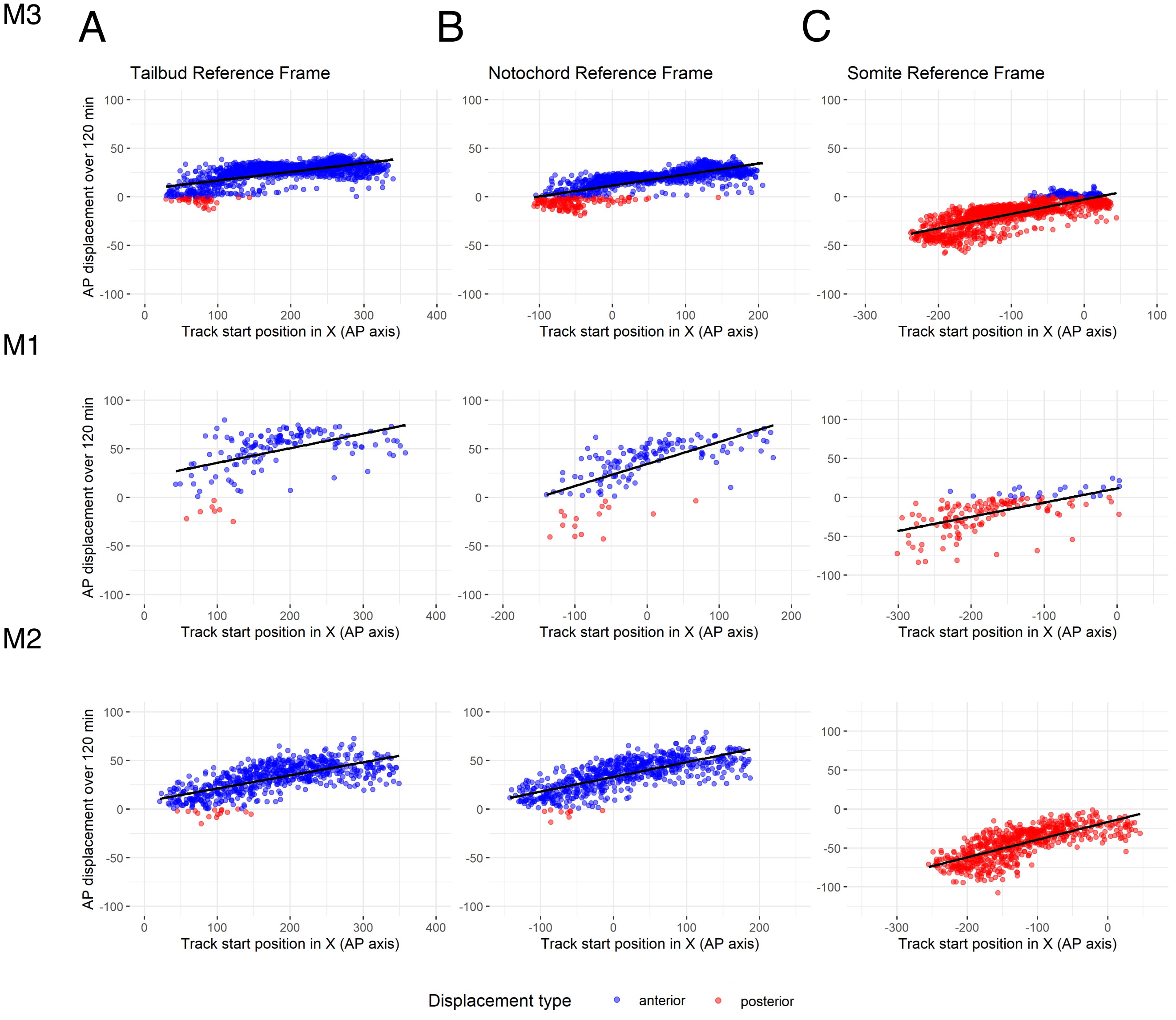
M3
A
B
C
Tailbud Reference Frame
Notochord Reference Frame
Somite Reference Frame
AP displacement over 120 min
Track start position in X (AP axis)
M1
M2
Displacement type
anterior
posterior

Figure S8
