# Supplementary material for: The zebrafish presomitic mesoderm elongates through compaction-extension
Source: Cells Dev. Author manuscript; Available in PMC 2022 May 11. (PMC7612712; doi:10.1016/j.cdev.2021.203748)
Supplement: Supplementary [file EMS140703-supplement-Supplementary.docx]

## Supplementary figure legends

**Figure S1: Tissue reconstructions of the PSM and somites.**

(A) 2D contours (yellow outlines) manually drawn around the PSM at regular z-slices, up to the midline. (B) Maximum projections showing all PSM contours, from lateral, dorsal, and posterior views (left to right). (C) Same images as in B but showing PSM surface (cyan). (D) Contours and surface (pink) of the nascent somite (left, middle), and automatic *msgn1* surface (gold, right). All images are of the same 23 somite-stage embryo. Scale bar: 50 µm.

**Figure S2: Cells are lost from the paraxial mesoderm during somitogenesis.** *h2a*::mCherry (red) x *tbx16*::GFP (cyan) were live-imaged for the latter stages of somitogenesis. Left images show both nuclei (red) and *tbx16*::GFP (cyan), while right images show only *tbx16*::GFP. Cells can be seen moving out of the somites and PSM into the dorsal (white arrow) and ventral (yellow arrow) fin mesenchyme. All images are maximum projections. Scale bar: 40 μm.

**Figure S3: Two-photon time-lapse imaging of the zebrafish tailbud.**

Three embryos of different stages were live-imaged for 2-3 hours each. Nuclei are labelled with either *h2a*::mCherry or *h2b*::GFP (both shown as grey). Images are shown of the tailbud at 0, 1, and 2 hours after the start of imaging. All images are maximum projections. Scale bars: 40 µm. Each movie is summarized in the table below, in terms of somite stages, frame interval, and nuclear label.

**Figure S4: Normalizing cell movements for global tail uncurling.**

A “reference frame” was placed at the DV/ML midline of the tailbud tip (with axes lined up to match biological axes) at regular intervals. (A) Individual z-slice showing reference frame placement over 2 hrs. Scale bar: 50 µm. (B) Images and cell tracks before (left) and after (right) “resampling” images to fix the reference point (tailbud tip) in space. Images and tracks (all cells) show a large dorsal movement before resampling, but none after resampling. Tracking colour code (blue to red) indicates time. Scale bars: 40 µm. All images from M3. Anterior is top, dorsal is left.

**Figure S5: Validation of automatic tracks.**

(A) Tracks were generated of the whole tailbud (first hour of movie M3) using 5 different algorithms: Autoregressive Motion (AM); Autoregressive Motion Expert (AME); Brownian Motion (BM); Connected Components (CC); and Lineage (LI). For each algorithm, two sets of tracks were made for two Max Distance (MD) parameters (5, 10). Boxplots show track duration for each set of tracks. AME and CC were excluded from further analysis due to short track durations. (B) Manual validation of track accuracy (LI was excluded at the beginning of analysis due to high false positive reporting of cell divisions). Tracks were generated for AM and BM, for three MD parameters each (5, 7.5, 10), and 20 PSM tracks (with duration > 60 min) were randomly selected for validation over 60 min. Number of errors (top left) and number of accurate tracks (error-free tracks) (top right) were recorded. Track gaps were also recorded (bottom left), and the percentage of accurate (error-free) gaps were noted (bottom right). (C) Having identified AM as the best algorithm, and MD = 5 as the best value for movie M3, tracks were made for all three full movies. For M1 and M2, two sets of tracks were made with an adjusted (for difference in frame interval) and non-adjusted MD value. Boxplot shows track duration for each set of tracks. For each track set, 20 PSM cells were randomly selected and validated as before. Bar charts show number of errors (left) and number of accurate tracks (right). (For M1, the adjusted MD tracks were not validated, given the perfect accuracy and longer duration of non-adjusted MD tracks.)

**Figure S6: Neighbourhood analysis.**

Analysis was performed using MATLAB scripts written by L.M. (A) Neighbourhood analysis results for all three movies (left to right), over a time period of 120 min, for small (k = 10, top row) and large (k = 50, bottom row) neighbourhoods (note different y-axis scales). Each point represents the neighbourhood of one cell. The number of new cells which entered that cell’s neighbourhood is plotted against the starting position (AP) of that cell (0 = posterior). The results show a strong gradient of cell mixing, from the posterior (high mixing) to the middle (low mixing) of the paraxial mesoderm. From the middle to the anterior, there is a slight increase, likely related to somite morphogenesis. These results are highly similar to those of photolabelling experiments, confirming the reliability of cell tracks. (B) Track duration plotted against track start position, for each movie, to confirm that these patterns are not an artefact of track durations.

**Figure S7: Multiple biological reference frames (RFs) along the AP axis.**

(A) Individual z-slice showing tailbud (TB) RF as previously described (Figure 17); notochord (NC) RF placed at the posterior end of the notochord proper (where the notochord “funnels out” into the notochord progenitors); and somite (SOM) RF placed at the posterior boundary of the start-of-movie nascent somite. Time is shown from left to right. (B) All three RFs shown together on maximum projection images from lateral (top) and dorsal (bottom) views. Scale bars: 40 µm. All images from two-photon movie M3. Anterior is top, dorsal is left (except bottom row in B, in which medial is left).

**Figure S8: Anteroposterior displacement is relative to the reference frame.** Measurements of AP displacement of each track over 120 min are shown for all three movies (top to bottom), relative to three different reference frames: tailbud tip (A); notochord proper end (B); and the start-of-movie nascent somite (C). AP displacement is shown on the y-axis, with positive values indicating anterior displacement, and negative values indicating posterior displacement (points are also colour coded for displacement type to highlight this). This is plotted against track start position, where 0 is the reference frame position (negative values are posterior to this, positive values are anterior). The data shows that almost all cells move anteriorly relative to the tailbud tip, but also that almost all cells move posteriorly relative to the nascent somite, with displacement relative to the notochord showing similar results to that of the tailbud tip, but with slightly more posterior displacement. All trendlines (black) show a positive correlation i.e. anterior cells move anteriorly while posterior cells move posteriorly.

## Movie captions

**Movie S1: 3D morphometric measurement workflow.**

*in situ* hybridization chain reaction (HCR) for *msgn1* (yellow) and *tbx6* (red), with nuclei labelled by DAPI staining (grey). Image is a rotating 3D view of a confocal image. Each z-slice is then shown (scrolling from lateral to medial and back). Manual contours (cyan lines) were drawn around the PSM up to the embryo midline. A 3D reconstruction was generated from these contours (cyan surface), providing tissue volume information. DAPI signal was then isolated from this surface and used to generate a spot of each nucleus centre (cyan spheres), providing cell number information. Measurements of height and width of both the posterior (yellow lines) and anterior (red lines) PSM, as well as the length of the PSM (white line). The movie shows this process for the PSM at the 23 somite-stage, but the same process was done for the PSM, and the nascent somite, at each somite stage from the 16 to the 32 somite-stage.

**Movie S2: Convergent extension of photolabels.**

Zebrafish embryos were injected at the one cell stage with mRNA for KikGR, a photoconvertible protein which localizes to the nucleus of each cell (shown in cyan). Dorsoventral stripes (red) along the length of the PSM were photoconverted with a UV laser on a confocal microscope. The movie shows a live zebrafish tailbud with three labels along the PSM. The move shows 2 hours of imaging (at 28^o^C), which then rewinds to show original label positions again. The posterior label (bottom right) undergoes far more convergent extension than the middle and anterior labels.

**Movie S3: Paraxial mesoderm cell tracks.**

*h2b*::GFP zebrafish embryos were live imaged on a two-photon microscope at 28^o^C. A reference frame was placed at the end of the tail at regular time intervals, and images were then registered to this point, to eliminate tail movement/uncurling before 3D automatic tracks of all nuclei were generated. All cell tracks outside the paraxial mesoderm were manually removed, to create a tracking of only paraxial mesoderm cells. The movie shows imaging over 2 hours, with paraxial mesoderm tracks colour-coded by AP position. At each timepoint, paraxial mesoderm tracks shown movement over the previous 5 minutes of imaging.
